# Genome-Wide Identification of Apple Atypical bHLH Subfamily PRE Members and Functional Characterization of MdPRE4.3 in Response to Abiotic Stress

**DOI:** 10.3389/fgene.2022.846559

**Published:** 2022-03-24

**Authors:** Tong Li, Yan Shi, Baihui Zhu, Tianen Zhang, Ziquan Feng, Xiaofei Wang, Xiuming Li, Chunxiang You

**Affiliations:** State Key Laboratory of Crop Biology, Shandong Green Fertilizer Technology Innovation Center, Collaborative Innovation Center of Fruit and Vegetable Quality and Efficient Production, Shandong Agricultural University, Tai’an, China

**Keywords:** *Malus domestica*, atypical bHLH, Paclobutrazol Resistance, abiotic stress, genome-wide identification

## Abstract

*Paclobutrazol Resistance* (*PRE*) genes encode atypical basic helix–loop–helix (bHLH) transcription factor family. Typical bHLH proteins contain a bifunctional structure with a basic region involved in DNA binding and an adjacent helix–loop–helix domain involved in protein–protein interaction. PRE members lack the basic region but retain the HLH domain, which interacts with other typical bHLH proteins to suppress or enhance their DNA-binding activity. PRE proteins are involved in phytohormone responses, light signal transduction, and fruit pigment accumulation. However, apple (*Malus domestica*) PRE protein functions have not been studied. In this study, nine *MdPRE* genes were identified from the apple GDDH13 v1.1 reference genome and were mapped to seven chromosomes. The *cis*-acting element analysis revealed that *MdPRE* promoters possessed various elements related to hormones, light, and stress responses. Expression pattern analysis showed that *MdPRE* genes have different tissue expression profiles. Hormonal and abiotic stress treatments can induce the expression of several MdPRE genes. Moreover, we provide molecular and genetic evidence showing that *MdPRE4.3* increases the apple’s sensitivity to NaCl, abscisic acid (ABA), and indoleacetic acid (IAA) and improves tolerance to brassinosteroids (BR); however, it does not affect the apple’s response to gibberellin (GA). Finally, the protein interaction network among the MdPRES proteins was predicted, which could help us elucidate the molecular and biological functions of atypical bHLH transcription factors in the apple.

## Introduction

The bHLH is a superfamily of transcription factors (TFs) widely found in animals and plants, named for its highly conserved basic/helix–loop–helix domain. It is the second largest TF family among eukaryotic proteins after v-myb, the avian myeloblastosis viral oncogene homolog (MYB) (Feller et al., 2011). The bHLH proteins contain a bifunctional structure with a basic region involved in DNA binding and an adjacent helix–loop–helix domain involved in homo- or hetero-dimerization (Li et al., 2006; Carretero-Paulet et al., 2010). Based on the DNA-binding ability, these proteins are divided into two major groups: DNA-binding bHLH (typical bHLH) and non-DNA-binding bHLH (HLH) proteins, also known as atypical HLH (Li et al., 2006). Atypical bHLH proteins lack the basic region; therefore, they cannot bind to DNA, but their HLH domain can interact with other typical bHLH proteins to suppress or enhance their DNA-binding activity (Li et al., 2006).

The PRE proteins belong to atypical bHLH transcription factors that have been intensively studied in recent years. They are involved in signal transduction pathways of hormones, temperature, and light responses and regulate plant growth and development in a variety of ways (Hyun and Lee, 2006; Tanaka et al., 2009; Wang et al., 2009; Zhang et al., 2009; Mara et al., 2010; Bai et al., 2012a; Oh et al., 2014; Zhu et al., 2017).

In *Arabidopsis thaliana*, six PRE genes are shown to have different functions in plant growth and development. PRE1/BANQUO1 ((BNQ1)/bHLH136) was initially identified as a positive regulator of the gibberellin (GA) response. The PRE1 transcription level is increased by GA signaling through GID receptor- and DELLA-dependent mechanisms, demonstrated in gibberellin-mediated seed germination, hypocotyl/petiole elongation, flower induction, and fruit development (Lee et al., 2006). Further studies showed that PRE1 is also involved in brassinosteroids (BR), indoleacetic acid (IAA), and light signaling (Zhang et al., 2009; Hao et al., 2012; Oh et al., 2012). Overexpression of PRE1 and its rice homolog lNCREASED LAMINA INCLINATION1 (ILI1) increased BR-induced cell elongation in both *Arabidopsis* and rice (Zhang et al., 2009). The PRE1 homologous protein in rice, BRASSINOSTEROID UPREGULATED 1 (BU1), participates in the BR signaling pathway to positively regulate bending of the lamina joint in rice (Tanaka et al., 2009). PRE3/bHLH135/ATBS1/TMO7 stimulates BR signaling by interacting with the BR negative regulator ATBS1-interacting factors (AIFs) and inhibiting their functions (Wang et al., 2009). PRE3 is a target gene for IAA response factor 5 (ARF5), required for rootstock development (Schlereth et al., 2010). In addition, PRE3 reduces photosensitivity by decreasing the response to red, far-red, and blue light and reduces lateral roots’ initiation (Castelain et al., 2012). PRE4 affects light-related physiological processes such as chlorophyll levels, sepal and carpel color, and early and late flowering (Mara et al., 2010). PRE6 is a transcriptional repressor that negatively regulates IAA response (Zheng et al., 2017). It is involved in light signal transduction, and its gene expression is regulated by light (Hao et al., 2012). Habitually, it is common for multiple PRE genes to have overlapping functions. PRE3 and PRE6 actively regulate organ elongation by interacting with other bHLH proteins, including AIFs and LONG HYPOCOTYL IN FAR-RED1 (HFR1) (Hyun and Lee, 2006; Wang et al., 2009). Both PRE2 and PRE6 are abscisic acid (ABA) sensitive genes involved in plant growth regulation and environmental stimulation (Zheng et al., 2019). PRE1, PRE4, and PRE6 are direct targets of BRASSINAZOLE-RESISTANT1 (BZR1) and phytochrome-interacting factor 4 (PIF4), induced by BR, GA, and high temperature and inhibited by light. Suppressing PRE1, PRE2, PRE4, and PRE6 leads to dwarfism and hyposensitivity to BR, GA, and high temperature and hypersensitivity to light (Bai et al., 2012; Oh et al., 2012; Oh et al., 2014). PRE1, PRE2, and PRE4, which play a role in flower development, are direct target genes of APETALA3/PISTILLATA (AP3/PI) negative regulation in petals (Mara et al., 2010).

Current research on *PRE* genes in plants focuses on *Arabidopsis thaliana*, where PREs are involved in cell elongation, hormone signaling regulation, photomorphogenesis, and other important growth and development pathways (Lee et al., 2006; Zhang et al., 2009; Bai et al., 2012a; Castelain et al., 2012). To explore the functions of the PRE-related genes in apple growth and development and response to environmental stress, a total of nine members of the PRE subfamily were identified in apple through homologous sequence alignment. To date, no systematic studies on the apple atypical bHLH gene subfamily, *MdPREs*, have been reported. Given the importance of atypical bHLH subfamilies (e.g., PREs) in plants, this study performed a genome-wide analysis of *MdPREs* using the GDDH13 v1.1 reference genome of the diploid “Golden Delicious” apple (Daccord et al., 2017). Moreover, the *MdPRE* gene family characterization was performed using bioinformatics and molecular biology methods, such as genetic structure, promoter analysis, expression pattern, chromosome localization, apple callus transformation, and protein–protein interaction prediction. This study lays the foundation for clarifying the biological and molecular functions and evolutionary diversity of atypical bHLH transcription factors in *Malus domestica*.

## Materials and Methods

### Genome-Wide Identification of the *PRE* Genes in *Malus domestica*

The *Malus domestica* genome used in this study was the “Golden Delicious” apple GDDH13 v1.1 reference genome (GDDH13_1-1,https://iris.angers.inra.fr/gddh13/) (Daccord et al., 2017). Blastp was used to identify all MdPRE members. *A. thaliana* PRE protein sequences with sequence numbers referenced from Siefers *et al.* and downloaded from the TAIR database (https://www.Arabidopsis thaliana.org/) (Siefers et al., 2009) were used as the query sequence for protein homology alignment. The six AtPRE sequences are AtPRE1 (At5g39860), AtPRE2 (At5g15160), AtPRE3 (At1g74500), AtPRE4 (At3g47710), AtPRE5 (At3g28857), and AtPRE6 (At1g26945). The searched apple members were submitted to SMART (http://smart.embl-heidelberg.de/) for conservative structural domain confirmation (Letunic and Bork, 2018), resulting in the candidate *MdPREs.*

The *MdPRE* gene length was obtained from the GFF3 annotation file of the “Golden Delicious” apple GDDH13 v1.1 reference genome. The length, isoelectric point (*pI*), molecular weight, and charge at pH 7.0 of the PRE protein sequences were predicted using DNAstar software (DNASTAR 7.1, http://www.dnastar.com). The bHLH structural domain position of MdPREs was analyzed using the Pfam database.

### *MdPRE* Chromosomal Localizations and Gene Structures

The chromosome localization information of apple *PREs* was downloaded from the GDR database (https://www.rosaceae.org/; gene_models_20170612.gff3). *MdPRE* chromosomal localization was mapped using MG2C (http://mg2c.iask.in/mg2c_v2.1/) (Chao et al., 2015). GSDS 2.0 service (http://gsds.gao-lab.org/index.php) was used to analyze the exon–intron structure of *MdPREs* (Hu et al., 2015).

### Structural Domains, Evolutionary Genetic Analysis, and Protein Structure Prediction of *MdPREs*

Clustal Omega (https://www.ebi.ac.uk/Tools/msa/clustalo/) was used to perform multiple sequence alignment and structural domain analysis of MdPRE protein sequences, and the results were visualized in Jalview 2.11.1.4 (Waterhouse et al., 2009). Genetic evolution analyses of maximum likelihood and neighbor-joining trees (MdPREs) were performed in MEGA_X with the step test set to 1,000 times (Kumar et al., 2018). The PRE sequences of *Arabidopsis thaliana* were referenced from Petroni et al. (2012). Based on the available literature, a combination of SGN (Solanaceae Genomics Network, solgenomics.net, Zhu et al., 2017), RGAP (Rice Genome Annotation Project, rice.uga.edu, Guo Pengyu, 2021), and COTTONGEN (www.cottongen.org, Zheng, 2020) databases, a total of thirty-seven related genes were found for five PREs in tomatoes, seven ILIs in rice, and twenty-five PREs in cotton.

For the conserved motif analysis of *MdPRE* genes, the MEME5.1.1 server (http://memesuite.org/tools/meme) was used (Bailey et al., 2009), where the Zoops site distribution was chosen, and the maximum module length was set to 60.

The 3D structure analysis of MdPRE proteins was performed using the homology modeling service Phyre^2^ (http://www.sbg.bio.ic.ac.uk/phyre2/html/page.cgi?id=index) (Kelley et al., 2015).

### *MdPRE* Promoters Analysis

The entire apple genome sequence was downloaded from the GDR database, and the 2.0-kb-long sequences upstream of the transcription start site of the nine *MdPRE* genes were extracted. The *cis*-acting elements related to stress responsiveness and plant hormones in the promoter regions of the *MdPRE* genes were analyzed using PlantCARE (http://bioinformatics.psb.ugent.be/webtools/plantcare/html/) software. The results were visualized using TBtools (V 1.068; https://github.com/CJ-Chen/TBtools).

### Protein Association Network Prediction

The interaction network of MdPRE proteins in the apple was predicted using the online STRING database (version 11.5; http://stringdb.org), using *Arabidopsis thaliana* as the specified organism. The network was constructed using the method described by Mao et al. (2017).

### Plant Materials and Growth Conditions

In 2020, different apple tissues (primary roots, annual shoots, fully developed mature leaves, flowers at the first flowering stage, and fruits 150 days after flowering) were obtained from “Royal Gala” apple trees at the Experimental Station of Shandong Agricultural University, immediately frozen in liquid nitrogen, and stored at −80°C for studying the expression pattern of *MdPREs*.

[*Malus hupehensis* (Pamp.) Rehd. pingyiensis] seeds were collected from the experimental station of Shandong Agricultural University. The seeds and sand were soaked in a low-concentration potassium permanganate solution for 2 h for surface disinfection. Then the seeds and wet sand were uniformly mixed and laminated and then stored in a refrigerator at 4°C for 45 days. *M. hupehensis* laminated seeds were planted in vermiculite cavity trays for about 75 days. When *M. hupehensis* seedlings had about 7-8 true leaves, they were transferred to water for 5 days. The lower part of the ground of the seedlings at the same growth state was treated with various hydroponic solutions: NaCl (100 mmol L^−1^), ABA (150 μmol L^−1^), IAA (50 μmol L^−1^), BR (15 nmol L^−1^), and GA (100 μmol L^−1^), sampled after 0, 1, 2, 3, 6, and 12 h. After treatment, whole seedlings were immediately frozen in liquid nitrogen and stored at −80°C for subsequent analysis.

*Malus domestica* “Orin” apple calluses were grown on MS medium with 0.45 mg L^−1^ 6-BA, 1.6 mg L^−1^ 2,4-D, 20 g L^−1^ sucrose, and 6.0 g L^−1^ agar powder and adjusted to pH 5.9 with 1.0 mol L^−1^ sodium hydroxide. Calluses were cultured in the dark at 26°C and subcultured every 18 days.

### Quantitative Real-Time PCR Analysis

RNA was extracted from apple tissues using the RNA Plant Plus kit (TIANGEN, Beijing, China), and cDNA was obtained by using the PrimeScript RT reagent kit with gDNA Eraser kit (TaKaRa, Dalian, China). The iCycler iQ5 System (Bio-Rad) was used for quantitative real-time PCR assays. The 2^−ΔΔCt^ method was used to analyze the data. In addition, three independent replicates were also performed. *Md18s* was used as an internal reference gene. The sequences of the primers used for quantification are shown in Supplementary Table S1.

### Construction of the *MdPRE4.3* Expression Vector and Genetic Transformation Into Apple Callus

The full-length fragments of *MdPRE4.3* were amplified from *Malus domestica* “Gala” apple using the polymerase chain reaction (PCR). The primers used were *MdPRE4.3*-F 5′ATG​TCA​AGT​AGA​AGA​CCA​TCA3′ and; *MdPRE4.3*-R 5′ATG​CTG​CAA​AAG​TCT​TCT​AA3’.

The full-length DNA fragment of *MdPRE4.3* was cloned into the pCAMBIA1300-cLuc plant expression plasmid downstream of the cauliflower mosaic virus (CaMV) 35S promoter. Subsequently, the pCAMBIA1300-cLuc plasmid and resulting constructs were transformed into *Agrobacterium tumefaciens* strain LBA4404 using the heat-shock method.

The wild-type (WT) and overexpressing (OE) *MdPRE4.3* transgenic apple callus were obtained using the *Agrobacterium*-mediated transformation method (Zhao et al., 2016).

### Apple Callus Growth Under the NaCl, ABA, IAA, BR, and GA Treatments

The 18-day-old WT and OE *MdPRE4.3* transgenic apple callus were subcultured on medium containing 100 mmol L^−1^ NaCl, 150 μmol L^−1^ ABA, 50 μmol L^−1^ IAA, 15 nmol L^−1^ BR, and 100 μmol L^−1^ GA, respectively, for 21 d in the dark. Growth was monitored using a fresh weight assay.

### Statistical Analysis

Three technical replicates with three biological replicates each were performed per experiment. Analysis of variance (ANOVA) was performed using SPSS. DPS software was used for significant difference analysis, and a *p*-value < 0.05 was considered a significant difference (*, *p* < 0.05; **, *p* < 0.01; ***, *p* < 0.001). Error bars represent the standard deviation.

## Results

### Identification and Characterization of Apple *MdPRE* Genes

Apple has 188 reported bHLH genes (Mao et al., 2017). To identify PRE members in apples, BLASTp analysis was performed using *A. thaliana* PRE protein sequences, and a total of nine MdPRE members were identified in the GDDH13 v1.1 reference genome (Table 1). They were named *MdPRE2.1*, *MdPRE2.2*, *MdPRE3.1*, *MdPRE3.2*, *MdPRE4.1*, *MdPRE4.2*, *MdPRE4.3*, *MdPRE6.1*, and *MdPRE6.2*, based on their homology with the *A. thaliana* PRE genes (Table 1). Sequence analysis showed that the predicted molecular weights of the MdPRE proteins ranged from 10229.41 (MdPRE3.2) to 11023.27 (MdPRE6.2) Da. Their predicted *pI* values ranged from 6.06 (MdPRE4.1) to 9.18 (MdPRE3.2).

**TABLE 1:** Information about the PRE members found in apples.

Gene name	Gene ID (2017)	Gene ID (2010)	Chromosome location	Position	Molecular weight (Da)	PI	Best hits	TAIR description	Score	E-value
*MdPRE2.1*	MD17G1049300	MDP0000799392	Chr17	3602274–3603946	10271.66	7.93	At5g15160	AtPRE2	114	6e-25
*MdPRE2.2*	MD09G1049300	MDP0000320691	Chr09	3280330–3281295	10345.8	9.03	At5g15160	AtPRE2	114	6e-25
*MdPRE3.1*	MD06G1190200	MDP0000738505	Chr06	32652522–32661143	10398.63	9.17	At1g74500	AtPRE3	225	4e-58
*MdPRE3.2*	MD14G1197100	MDP0000228273	Chr14	28717166–28718073	10229.41	9.18	At1g74500	AtPRE3	225	4e-58
*MdPRE4.1*	MD06G1190900	MDP0000545428	Chr06	32722191–32723627	10353.71	6.06	At3g47710	AtPRE4	56.3	5e-07
*MdPRE4.2*	MD14G1197600	MDP0000260125	Chr14	28776686–28781087	10450.75	6.41	At3g47710	AtPRE4	56.3	5e-07
*MdPRE4.3*	MD00G1186500	MDP0000204989	Chr00	44302692–44303097	10866.22	6.41	At3g47710	AtPRE4	56.3	5e-07
*MdPRE6.1*	MD16G1075700	MDP0000174388	Chr16	5303469–5304145	10976.26	9.09	At1g26945	AtPRE6	114	6e-25
*MdPRE6.2*	MD13G1074300	MDP0000210979	Chr13	5249066–5249714	11023.27	6.58	At1g26945	AtPRE6	114	6e-25

### Chromosome Localization and Gene Structural Analysis of the *MdPRE* Genes

**FIGURE 1:**
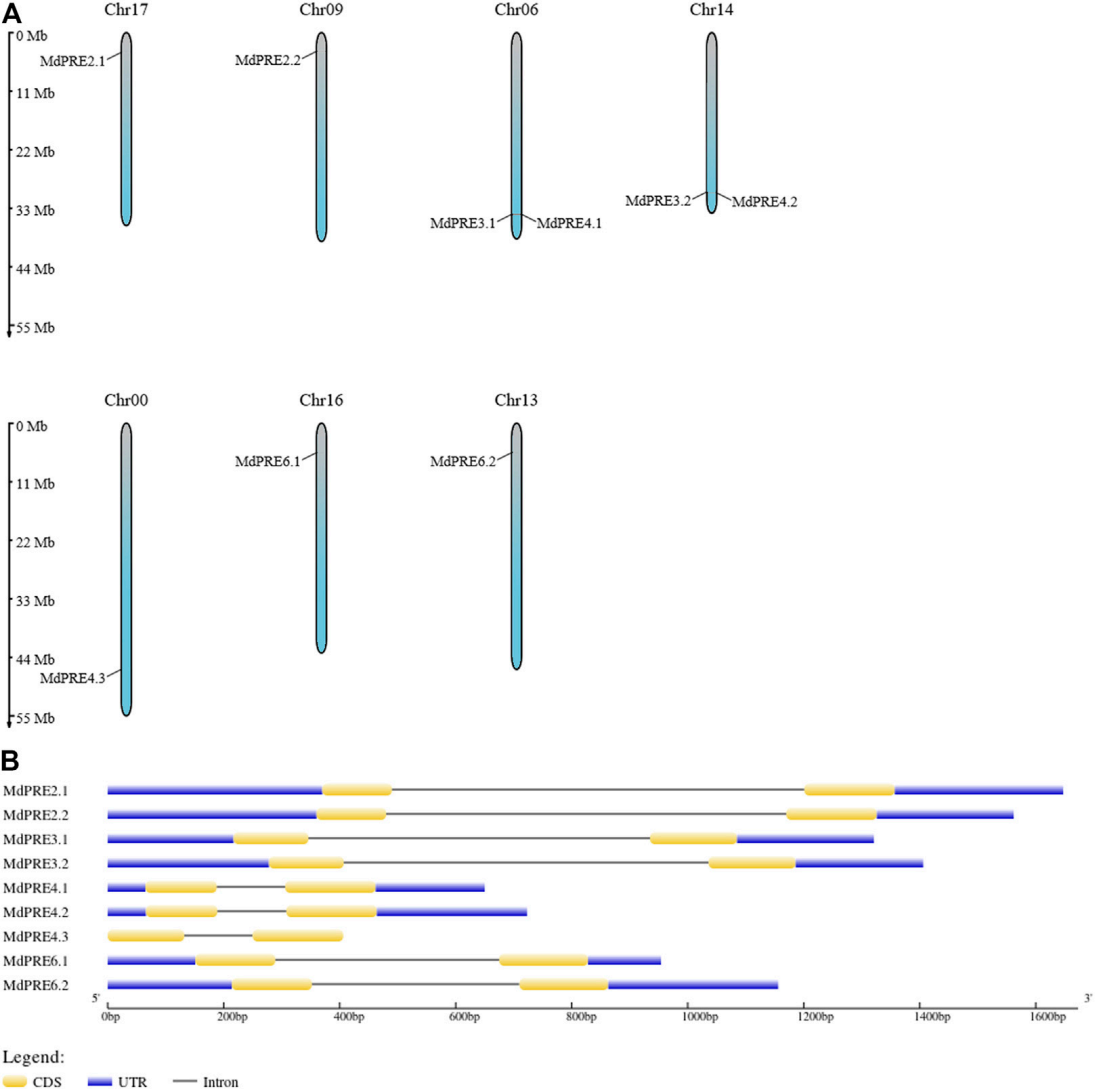
Chromosomal location and gene structure (intron/exon) of the *MdPRE* genes. **(A)** Chromosomal location of nine *MdPRE* genes on seven apple chromosomes. **(B)** Gene structures of the *MdPRE* genes.

*MdPRE* genes were mapped to seven chromosomes by analyzing genomic location information obtained from the GDR database. Chromosomes 00, 09, 13, 16, and 17 each contain one *MdPRE* gene, while chromosome 06 (containing *MdPRE3.1* and *MdPRE4.1*) and chromosome 14 (containing *MdPRE3.2* and *MdPRE4.2*) contain two *MdPRE* genes (Figure 1A). IDs and genomic positions of the identified MdPRE genes are summarized in Table 1.

To distinguish differences in the *MdPRE* gene structures, the exons and introns in the *MdPRE* gene sequences were analyzed. The analysis of the gene structures showed that the *MdPRE* gene coding region had a similar intron–exon gene structure, regardless of intron size. Except for *MdPRE4.3*, which does not contain a UTR, *MdPRE* genes contain two exons and one intron with similar distribution (Figure 1B), reflecting the relative stability of the MdPRE gene structure during evolution.

### Genetic Evolution and Protein Structure Analysis of MdPREs

**FIGURE 2:**
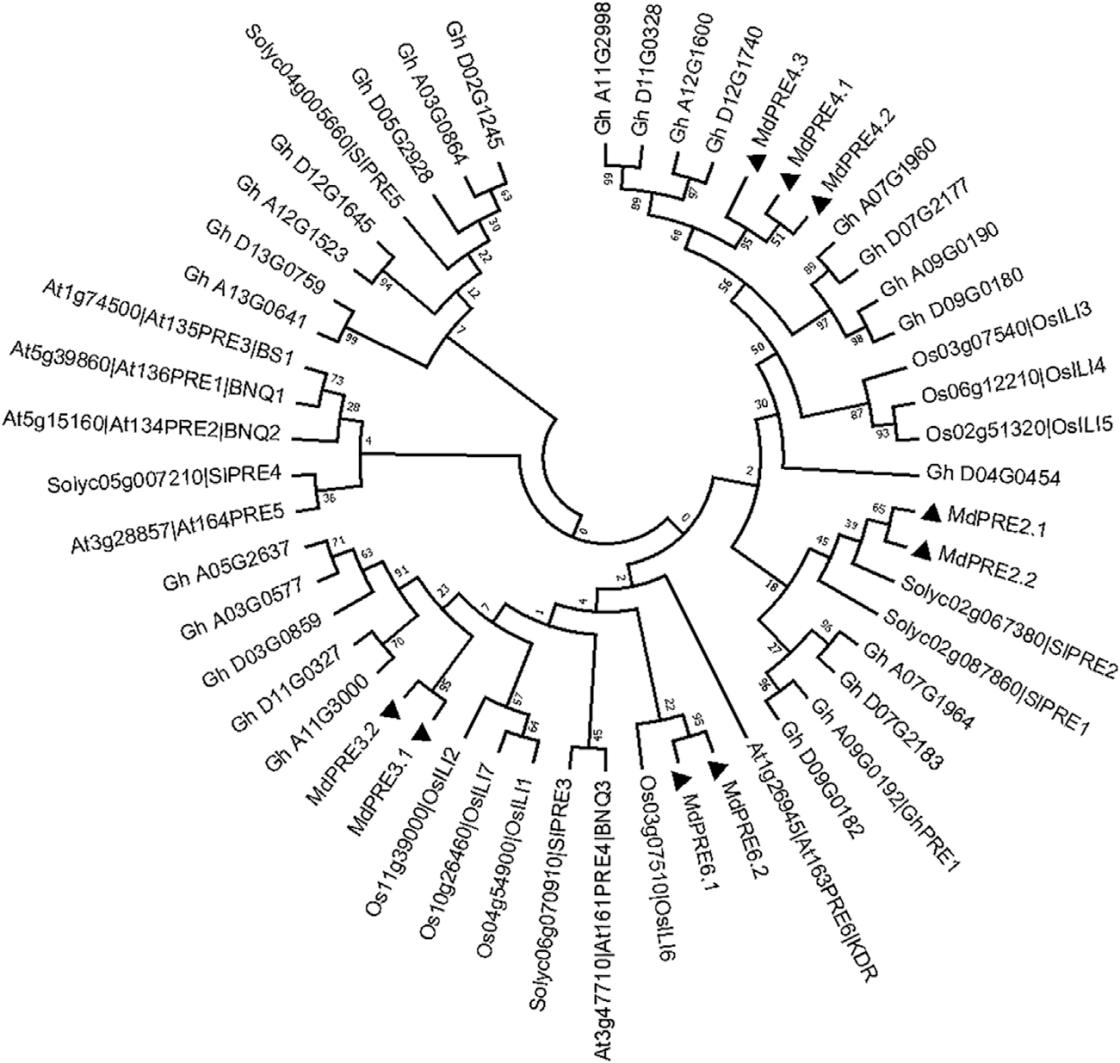
Evolutionary tree analysis of PRE proteins in apple, *Arabidopsis thaliana*, tomatoes, *Gossypium hirsutum*, and ILI proteins in rice (Maximum likelihood tree). Black triangle (▲) represents MdPRE protein.

To obtain the MdPRE taxonomic and evolutionary relationships, a maximum likelihood phylogenetic analysis was performed with all *A. thaliana*, tomato, *Gossypium hirsutum*, and ricePRE/ILI members, which shows that MdPRE2.1/2.2 are closely related to SlPRE2, MdPRE3.1/3.2/4.1/4.2/4.3 are more closely related to GhPREs, and MdPRE6.1/6.2 are closely related to OsILI6 (Figure 2). Subsequently, we named the family members of apple MdPREs based on their close relatives to the model plant *Arabidopsis thaliana*. This protein nomenclature for PREs has been applied to many other sequenced plants, such as tomatoes (SlPREs) (Zhu et al., 2017), strawberries (FaPREs) (Laura et al., 2019), and *Gossypium hirsutum* (GhPRE1) (Zheng, 2020).

**FIGURE 3:**
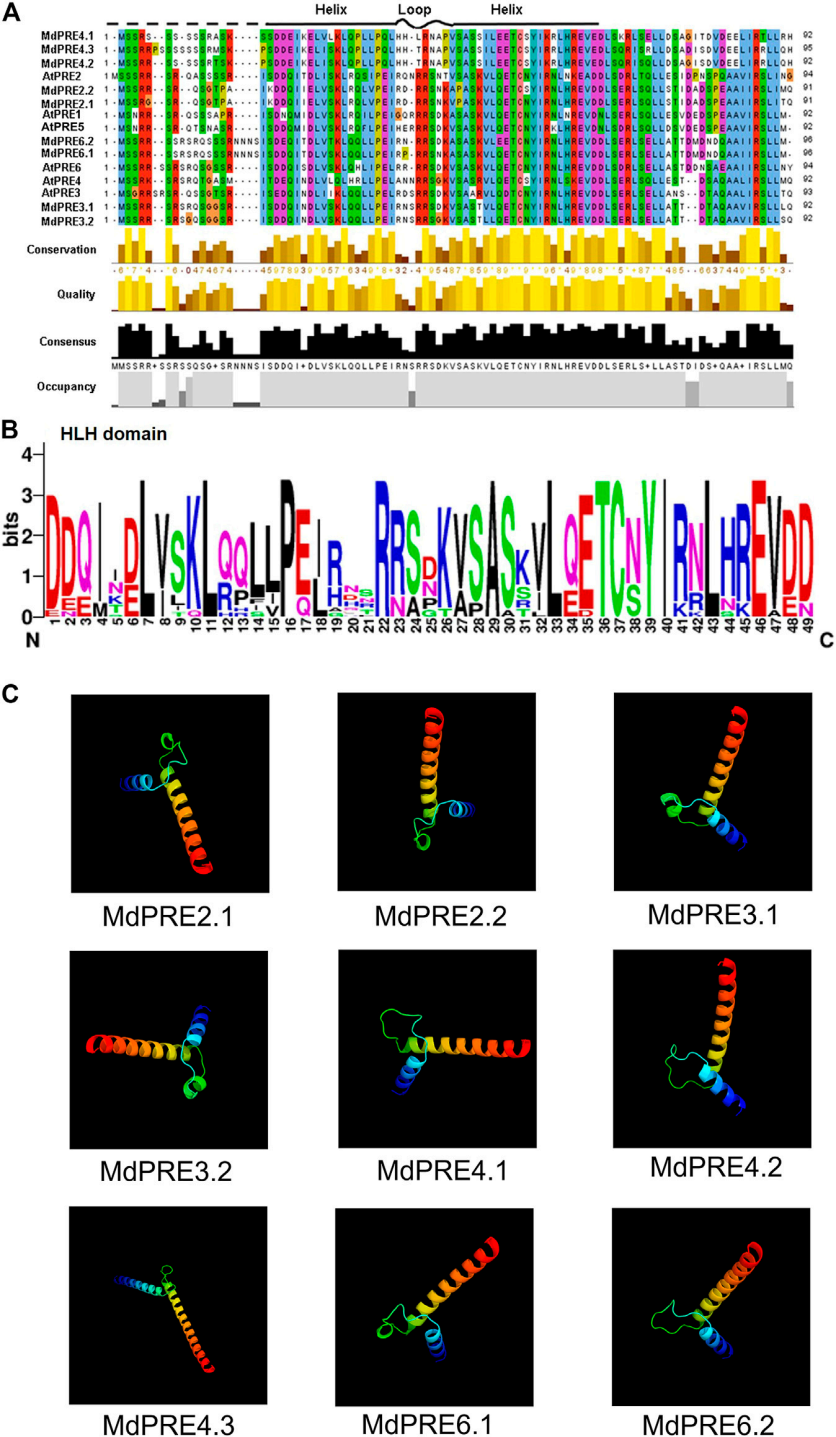
Multiple sequence alignment and conserved motif analyses of the MdPRE and AtPRE proteins. **(A)** The amino acid sequence alignment of the MdPRE and AtPRE proteins. Locations of the three conserved motifs are labeled with patterns. **(B)** The height of each letter shows the conservation of residues across the MdPRE and AtPRE proteins. The bit scores indicate the information content for each conserved motif in the sequence. **(C)** Three-dimensional structure of MdPRE proteins.

Figure 3A shows that MdPREs have a conserved structural domain, the helix–loop–helix (H-L-H) structural domain, but no typical basic structural domain. The HLH core conserved structural domain consists of three typical segments, which are two helix segments and one loop segment. The first α-helix is near the amino terminus and is the recognition helix, which recognizes and binds to specific protein sequences. The second α-helix is near the carboxyl terminus and is parallel to the double helix chain, and together with the first α-helix and the intermediate linker loop, they form the helix–loop–helix spatial structure (Murre et al., 1989). As shown in Figure 3B, all PRE members in apples and *A. thaliana* had the three typical HLH conserved regions, which constitute the core conserved structural domains of PRE, suggesting the functional conservation of MdPRE proteins. Then the high-level structure of MdPRE proteins was predicted using homology modeling. Figure 3C shows that the 3D structures of the nine MdPREs are similar. Moreover, the best templates exactly matched the core conserved domain region and showed a typical HLH structure, consistent with the *A. thaliana* results (Guo Pengyu, 2021).

### Expression Profiles of *MdPREs* in Different Apple Tissues

**FIGURE 4:**
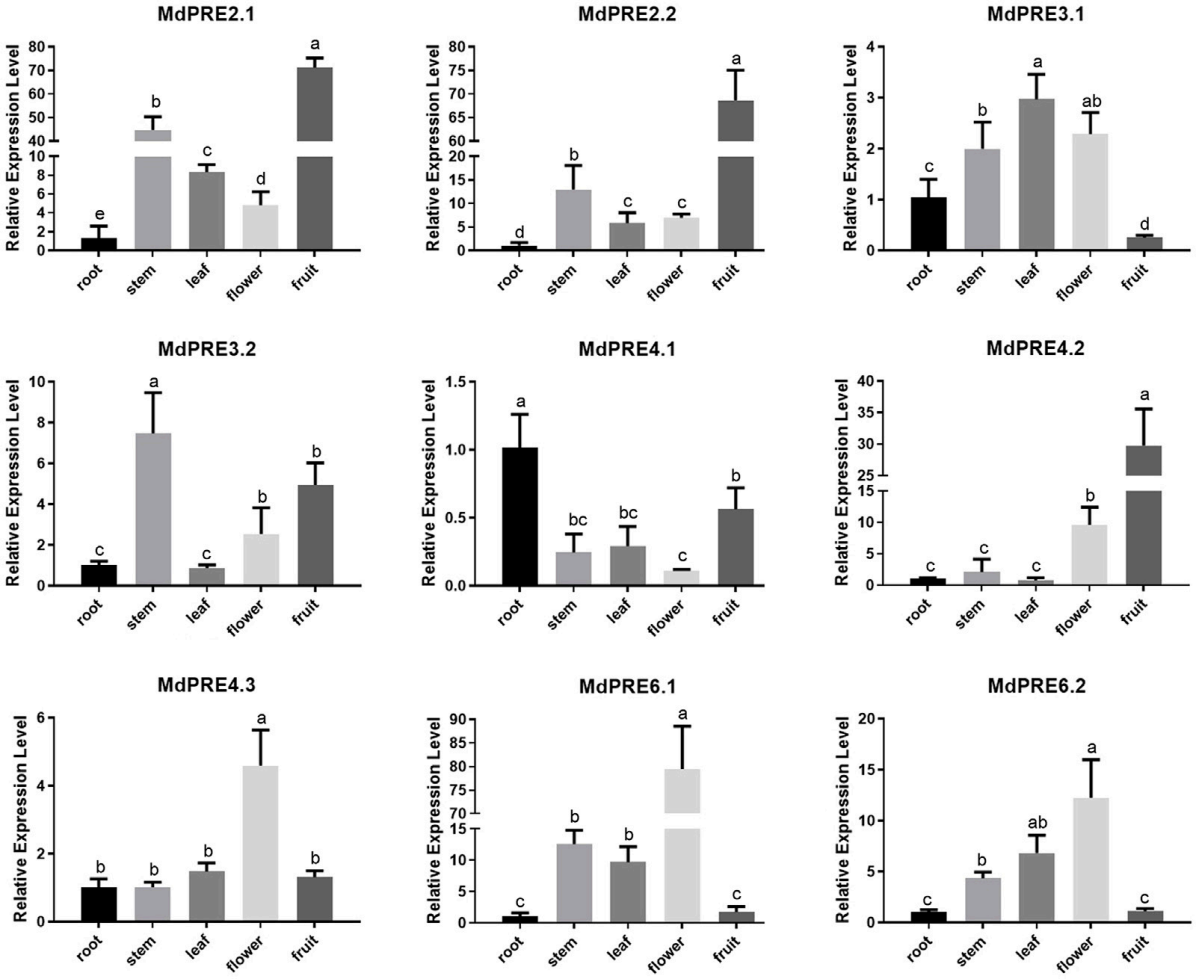
Relative expression analysis of the *MdPRE* genes in different tissues. Relative expression of the *MdPREs* was measured in apple primary roots, annual shoot, fully developed mature leaves, flowers at the first flowering stage, and fruits 150 days after flowering by qRT-PCR. The data were normalized to the expression of apple *Md18S.*

To initially investigate the temporal and spatial expression patterns of *MdPRE* genes in different tissues, *MdPRE* expression levels in primary roots, annual shoots, fully developed mature leaves, flowers at the first flowering stage, and fruits 150 days after flowering were examined using qRT-PCR (Figure 4). The results showed that the MdPRE gene family was expressed in all the tissues examined. *MdPRE3.1*, *MdPRE3.2*, and *MdPRE4.1* had the highest expression in leaves, stems, and roots, respectively, and they may be mainly involved in growth and development stages. Moreover, *MdPRE3.1* was significantly increased in all tissues tested, except for fruit. *MdPRE2.1*, *MdPRE2.2*, and *MdPRE4.2* had the highest expression in fruit. *MdPRE6.1/6.2* had the highest expression in flower, and *MdPRE4.3* was exclusively expressed in flower. Therefore, these genes might be mainly involved in the reproductive growth stage. *MdPRE2.1/2.2* in the stem, *MdPRE3.2* and *MdPRE4.1* in the fruit, and *MdPRE6.1/6.2* in the stem and leaf also showed high expression levels.

### Analysis of *cis-*Acting Elements in the Promoter of the *MdPRE* Genes

**FIGURE 5:**
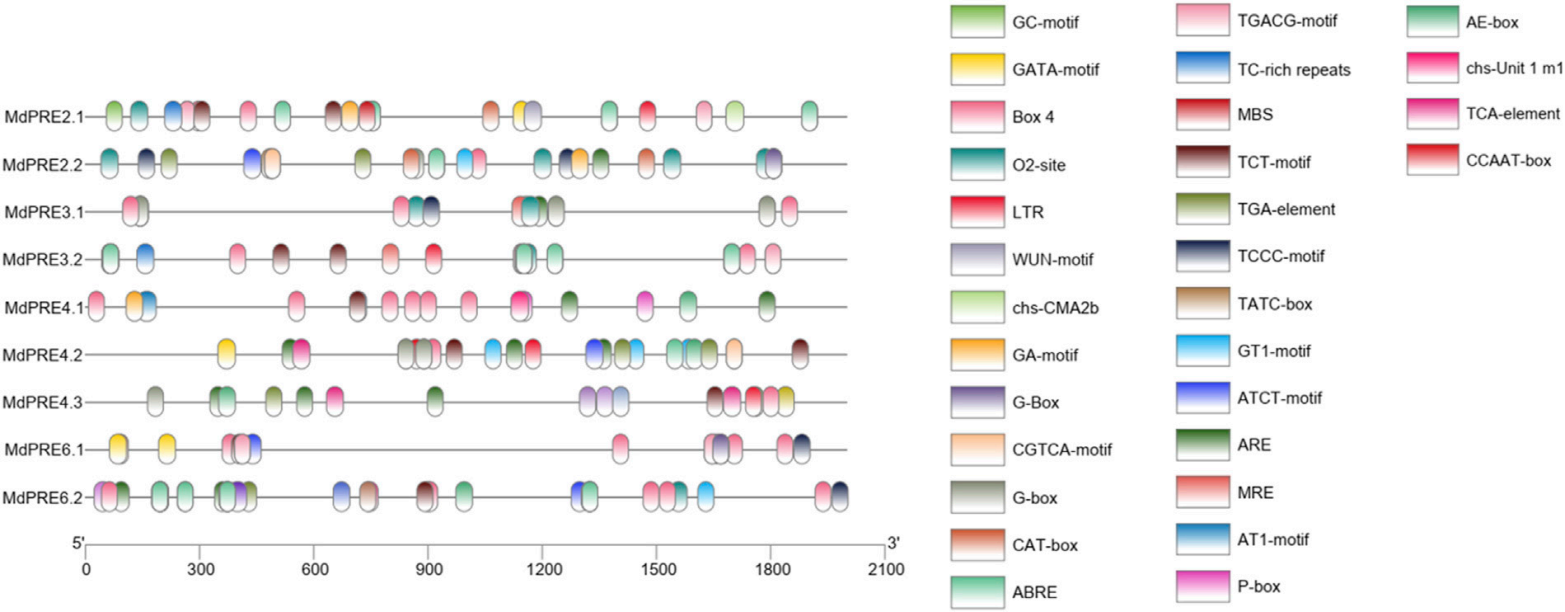
*Cis-*acting elements of *MdPRE* gene promoters. Promoter analysis was performed on 2000-bp sequences upstream of the transcription start sites.

To explore the potential regulatory factors of *MdPREs*, a prediction analysis of their promoter *cis*-acting elements was performed (Figure 5). The sequences 2000-bp upstream of each gene ATG were obtained from the GDR database, and *cis*-acting elements were analyzed by the online database PlantCARE website. The results showed many hormone-responsive elements (TGA-element, ABRE, CGTCA-motif/TGACG-motif, AuxRR-core, P-box, TCA-element) (Michal et al., 2019), stress-responsive elements (WUN-motif, TC-rich repeats ARE, LTR, MBS), and light-responsive elements (G-box, GA-box, GT1-motif, GATA-motif, Box4, TCT-motif) (Bastian et al., 2010; Porto et al., 2014; Ning et al., 2017). In addition, meristem expression elements (CAT-box) and tissue expression-specific regulatory elements (e.g., O2-site) were contained in some members, indicating that these genes may be influenced by multiple factors (An et al., 2019). For example, the *MdPRE4.3* promoter region contains three types of response elements mentioned earlier, and in addition, it contains HD-Zip1 (Sessa et al., 1993), a related element in fenestrated chloroplast differentiation. Taken together, the presence of these *cis*-acting elements suggests that *MdPREs* may be involved in multiple responses, which require further studies to elucidate.

### Expression Analysis of the *MdPRE* Genes Under Different Stress Conditions

**FIGURE 6:**
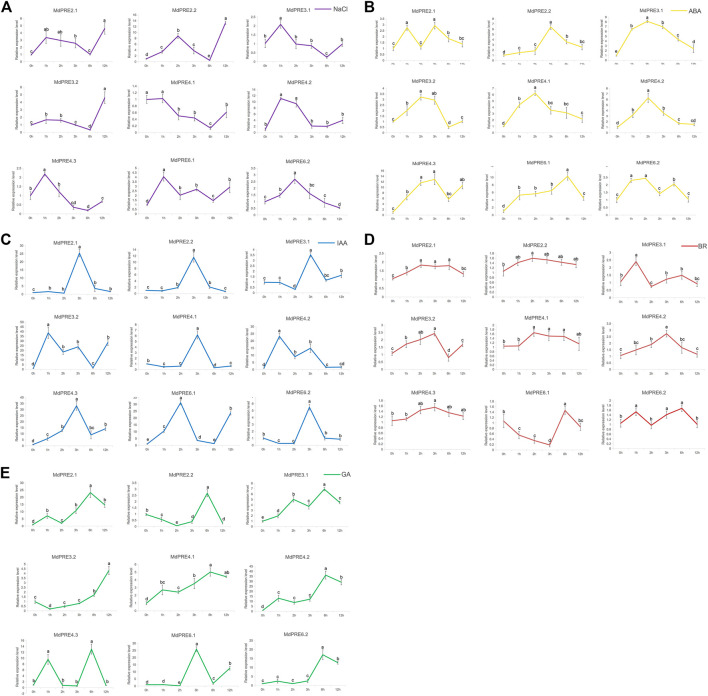
*MdPRE* genes relative expression analysis under different stress conditions. The expression levels under induced salt stress (**A**: NaCl; purple line), abscisic acid (**B**: ABA; yellow line), indole acetic acid (**C**: IAA; blue line), brassinosteroid (**D**: BR; red line) and gibberellin (**E**: GA; green line) of *MdPRE* genes measured by qRT-PCR analysis.

To investigate which of the nine *MdPRE* genes are significantly responsive to abiotic stresses and phytohormones, the changes in *MdPRE* transcript levels under NaCl, ABA, BR, IAA, and GA treatments were investigated. Consistent with the results of Figure 5, most *MdPRE* genes responded to different treatments. For example, the response pattern of *MdPRE* genes to NaCl treatment was complex and diverse, with most genes (*MdPRE2.1*, *MdPRE2.2*, *MdPRE3.1*, *MdPRE3.2*, *MdPRE4.2*, *MdPRE6.1,* and *MdPRE6.2*) showing upregulated expression levels after 1–2 h of treatment (Figure 6A). Under ABA (Figure 6B), IAA (Figure 6C), and BR (Figure 6D) treatments, the expression pattern of most *MdPRE* genes showed an increasing trend followed by a decrease. However, the highest expression level was reached at different time points for each gene. Except for Md*PRE4.3*, at the late stage of GA treatment, the expression levels of *MdPRE* genes were consistently significantly higher than the pretreatment expression levels (Figure 6E).

### *MdPRE4.3* Differential Tolerance/Sensitivity of Transgenic Apple Callus to NaCl, ABA, IAA, BR, and GA

**FIGURE 7:**
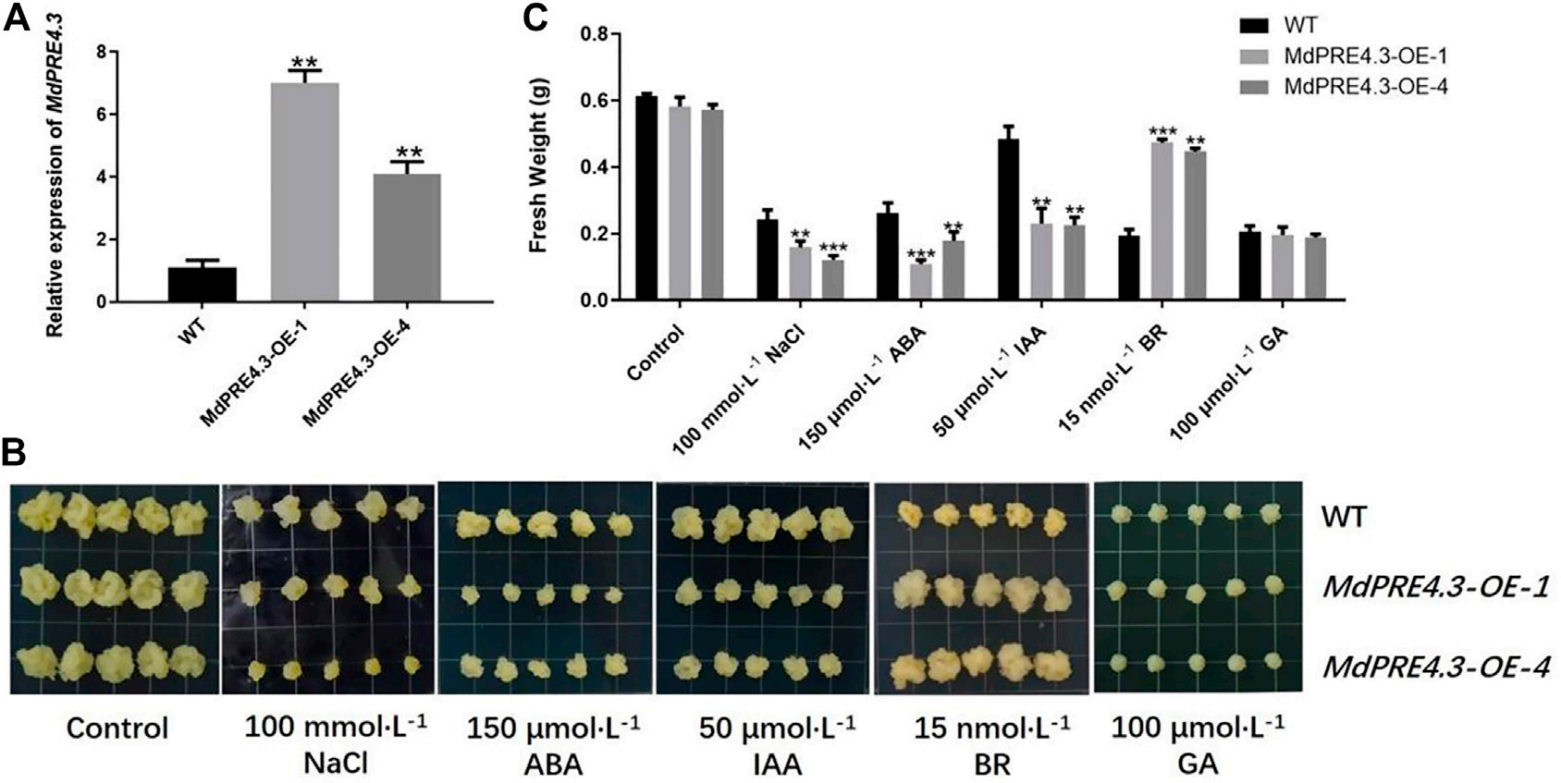
*MdPRE4.3* transgenic apple callus differential tolerance/sensitivity to NaCl, ABA, IAA, BR, and GA. **(A)** Expression analysis of *MdPRE4.3* in WT and *MdPRE4.3*-overexpressed transgenic calluses. **(B)** The phenotypes of WT and *MdPRE4.3*-overexpressing transgenic calluses treated with NaCl (100 mmol L^−1^), ABA (150 μmol L^−1^), IAA (50 μmol L^−1^), BR (15 nmol L^−1^), and GA (100 μmol L^−1^) for 21d, respectively. **(C)** Fresh weight in WT and *MdPRE4.3*-overexpressing transgenic calluses after treatment. Data are mean ± SD of three independent replicates.

The expression levels of most *MdPRE* genes were responsive to different stress conditions. To characterize the function of *MdPRE4.3* in apples, we obtained *MdPRE4.3* overexpressed transgenic apple calluses *MdPRE4.3-OE-1* and *MdPRE4.3-OE-4*. The transgenic callus generated much higher transcription levels of *MdPRE4.3* than the WT control, suggesting that *MdPRE4.3* was successfully transformed into the callus (Figure 7A). Under normal conditions, the fresh weight of *MdPRE4.3* overexpressed transgenic calluses was not significantly different from WT (Figures 7B,C). Under NaCl, ABA, and IAA treatment conditions, the fresh weight of *MdPRE4.3*-overexpressing calluses decreased more, especially under ABA treatment (Figure 7C). Under BR treatment, *MdPRE4.3*-overexpressing calluses exhibited a less sensitive phenotype than WT calluses (Figures 7B,C). The fresh weights of *MdPRE4.3*-overexpressing and WT calluses under GA stress treatment were significantly reduced (Figure 7B). However, the amount of their fresh weight reduction was not significantly different (Figure 7C).

### The Protein Interaction Network for the MdPRE Proteins Is Crucial for Growth Processes and Regulation

**FIGURE 8:**
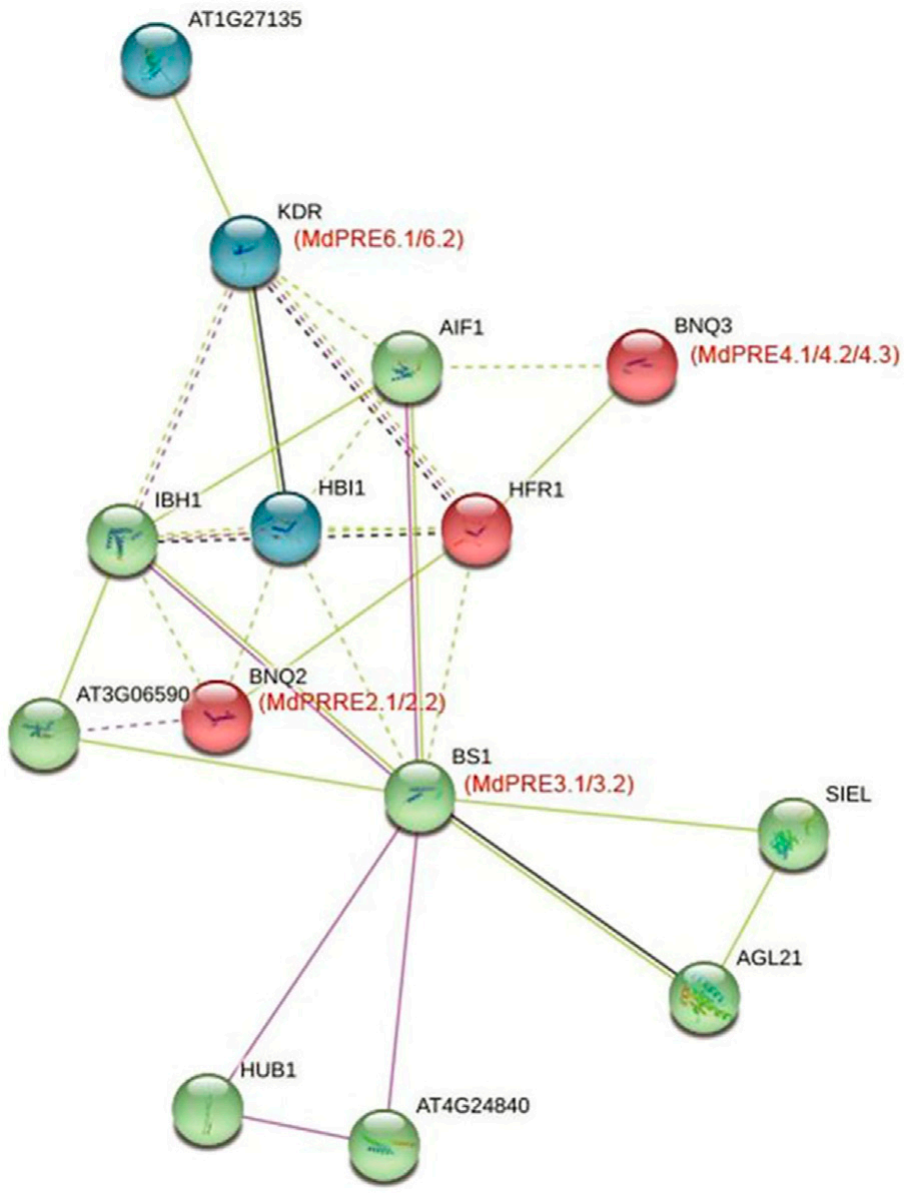
PREs protein interaction network in apples and *Arabidopsis thaliana*. This network was predicted using the online software STRING. MdPRE proteins are shown in brackets with the *Arabidopsis thaliana* orthologs.

The potential functions of apple PRE proteins were explored by mapping MdPREs to *A. thaliana* homologs using the protein–protein interaction database (Figure 8). In the protein function annotation step, MdPRE2.1/2.2 mapped to AtPRE2/BNQ2, MdPRE3.1/3.2 mapped to AtPRE3/BS1, MdPRE4.1/4.2/4.3 matched to AtPRE4/BNQ3, and MdPRE6.1/6.2 matched to AtPRE6/KDR. According to the predicted results, AIF1, HFR1, IBH1, and HBI1 interact with all PRE proteins and are involved in various biological functions, such as response to hormone signaling (Singh et al., 2017; Gruszka, 2018). In the network, AIF1 negatively regulates BR signaling (Ikeda et al., 2013); HFR1 is involved in phytochrome signaling (Gruszka, 2018); IBH1 negatively regulates cell and organ elongation in response to GA and BR signaling (Lu et al., 2018); and HBI1 acts as a positive regulator of cell elongation downstream of multiple external and endogenous signals. In addition, PRE proteins interact with HUB1 and AT3G06590 belonging to the BRE1 family in the network and are involved in the BR signaling pathway, respectively, indicating that PRE family members are closely related to BR signaling pathway members (Oh et al., 2014). SIEL and AGL21 are involved in plant root growth and developmental processes and interact with PRE3/BS1 (Li, 2010). This suggests that multiple protein interactions mediate the different regulatory processes involved in PREs.

## Discussion

Paclobutrazol Resistance (*PRE*) genes are a class of genes that encode proteins antagonistic to the GA synthesis inhibitor, paclobutrazol, which is a member of an atypical bHLH subfamily. Although PREs play an important role in plant hormone signaling and stress resistance, they have not been identified and functionally studied in apples. This study performed a systematic analysis and functional identification of the MdPRE family members through bioinformatics and plant genetic transformation. Combined with the existing reports of functional studies in other species, we provided directions for further studies of apple *PRE* genes and the selection of the genes for important traits.

To date, *PRE* genes have been identified in many other species, such as *AtPRE* genes in *Arabidopsis thaliana*, *SlPRE* genes in tomatoes (Zhu et al., 2017), *PRE* homologous genes *OsILIs* in rice, and *GhPRE* in *Gossypium hirsutum* divided into four subgroups: PRE-a, PRE-b, PRE-c, and PRE-d genes (Zheng, 2020). In this study, a total of nine MdPRE members were obtained in apples after rigorous screening and confirmation. Both maximum likelihood phylogenetic analysis and neighbor-joining phylogenetic analysis showed that MdPRE2.1/2.2 are closely related to SlPRE2, MdPRE3.1/3.2/4.1/4.2/4.3 are more closely related to GhPREs, and MdPRE6.1/6.2 are closely related to OsILI6. Overall, MdPREs might be closer in evolutionary distance to AtPRE2, AtPRE3, AtPRE4, and AtPRE6 (Figure 2; Supplementary Figure S1).

Generalization studies revealed that the basic amino terminal region of atypical bHLH proteins is loosely structured and lacks the necessary amino acids (Glu-13/Arg-17) for DNA binding; therefore, they do not act as transcription factors. However, these atypical bHLH proteins can form heterodimers with other bHLH transcription factors through the C-terminal HLH region and act as negative regulators of bHLH protein action, regulating downstream gene expression (Herold et al., 2002; Hernandez et al., 2007; Wang et al., 2009; Wei and Chen, 2018; Shin et al., 2019). A high degree of identity was found by comparing the core conserved domain loci of apple and *Arabidopsis thaliana*, especially in the two typical helix regions (Figure 3). This suggests that MdPREs may be evolutionarily close to AtPREs. A previous study comparing the PRE sequences of different species found that this functional region was highly conserved (Zheng, 2020; Guo Pengyu, 2021). This is supported by the homology modeling of the three-dimensional structures of MdPREs in this study (Figure 3C). Except for the consistency and conservativeness of the HLH core conserved structural domain region, there were large differences in other regions. The amino acid lengths of different PRE members in apples were highly variable. Similarly, the amino acid length of the ILI members of rice (87-130aa) is variable; in contrast, the PRE members’ amino acid length is relatively conserved in tomatoes (86-95aa) and *A. thaliana* (92-94aa) (Guo Pengyu, 2021). Analysis of the exon–intron structure of *MdPRE* genes shows that the coding regions of all genes consist of 5 and 3′ UTRs, two exons, and one intron. Except for *MdPRE4.3*, which contains only exons and introns, similar to the *OsILI3* and *OsILI4* genes in rice, this evidence suggests that *PRE* genes are evolutionarily stable and conserved in structure.

Apple’s *MdPRE* gene expression profiling revealed that different *MdPRE* members were differentially expressed in root, stem, leaf, flower, and fruit tissues, indicating that their spatial and temporal diversity is associated with their function in different tissues and at different growth and development stages (Figure 4). These results are consistent with those of previous studies in other plant tissues. In *Gossypium hirsutum*, *GhA09G0192* (*GhPRE1*), *GhD09G0182*, *GhA07G1964*, and *GhD07G2183* genes are abundantly expressed in the floral tissues (Zheng, 2020). In strawberries (*Fragaria ananassa*), *FaPRE1* is expressed almost exclusively in the ripe receptacle but not significantly in vegetative tissues (Laure et al., 2019b). In tomatoes (*Solanum lycopersicum Mill. cv. Ailsa Craig*), *SlPRE1* was specifically expressed in flowers, *SlPRE2* was highly expressed at 10 days after anthesis, *SlPRE3* was expressed in low abundance, *SlPRE4* was highly expressed in hypocotyl and vegetative tissues, and *SlPRE5* was expressed in multiple tissues (Zhu et al., 2017). In rice, *OsILI6* is expressed in the pistil, lemma, palea, and young panicle and predominantly in roots but not in leaves or the gynoecium (Heang and Sassa, 2012). Therefore, the different tissue expression patterns of *PREs* in different species suggest that they are involved in multiple biological processes and possess relatively complex functions.

There have been many studies on the regulatory mechanisms of *PREs* involved in hormonal signaling. *PRE1*, *PRE3*, *PRE4*, and *PRE6* expressions are induced by GA and BR, which positively regulate cell elongation by responding to the signaling pathways (Hyun and Lee, 2006; Lee et al., 2006; Wang et al., 2009; Mara et al., 2010). *PRE1*, *PRE3*, and *PRE6* are involved in IAA regulation, resulting in IAA-related growth phenotypes (Zhang et al., 2009; Schlereth et al., 2010; Zheng et al., 2017). In *Arabidopsis, PRE2* and *PRE6* are involved in the ABA-mediated regulation of salt response, and six *PRE* gene expression levels are reduced in response to ABA treatment but increased during salt treatment (Zheng et al., 2019). Similarly, in this study, *MdPRE* promoter analysis suggested that they all contain multiple *cis*-acting elements, including phytohormones, abiotic stresses, and light response elements (Figure 5). Simultaneously, a variety of hormones (ABA, IAA, BR, and GA) and abiotic stresses (such as NaCl) induced *MdPRE* expression, suggesting their function in response to stress resistance and growth processes in apples (Figure 6). The hormone combination in the control sample is the most suitable ratio for apple callus growth. On this basis, the addition of other hormones (such as ABA, IAA, BR, and GA) will have an adverse effect on its growth. Sequence comparison found extremely high similarities between *MdPRE* members, suggesting functional redundancy (Figure 3); thus, we cloned *MdPRE4.3* overexpressed transgenic callus for further experiments. The results showed that NaCl, ABA, and IAA treatment increased *MdPRE4.3* gene expression (Figure 6). Contrarily, *MdPRE4.3* overexpression in the callus showed high sensitivity to NaCl, ABA, and IAA compared with the WT callus (Figure 7), suggesting that MdPRE4.3 may be a positive regulator of these stress signaling pathways. GA treatment elevated *MdPRE4.3* gene expression levels (Figure 6); however, *MdPRE4.3*-overexpressing calluses did not respond to GA treatment (Figure 7), so MdPRE4.3 may not be involved in the GA signaling pathway. The *MdPRE4.3* gene expression level was elevated upon BR treatment (Figure 6), but *MdPRE4.3*-overexpressing calluses showed an insensitive phenotype to BR (Figure 7), suggesting that PRE4.3 may be a negative regulator of the BR signaling pathway. Similarly, AtPRE6 is negatively regulated in the IAA signaling pathway but positively regulated in the ABA and salt signaling pathways (Zhang et al., 2017; Zhang et al., 2019), and *FaPRE1* is repressed by IAA and activated by ABA, but its expression is unaffected by GA (Laura et al., 2019). Taken together, these studies suggest that different *PRE* genes may play both redundant and specific roles in different signaling pathways.

In summary, the *PRE* family genes of apples were exhaustively studied in this study. The *MdPRE* gene expression in apple under different tissues and stress conditions was analyzed by qRT-PCR, and the *MdPRE4.3* gene function was analyzed in detail by transgenic technology. These results have greatly improved our understanding of the *MdPRE* genes and provided rich resources for subsequent study of *PRE* family genes in apples and other plants.

## Conclusion

In this study, nine MdPRE genes were identified from the apple GDDH13 v1.1 reference genome, and they were mapped to seven chromosomes. The expression pattern analysis showed that MdPRE genes have different tissue expression profiles. The results showed that MdPRE promoters possessed various hormones and light and stress response elements. Moreover, hormonal and abiotic stress treatments induce the expression of several MdPRE genes. Moreover, we demonstrated by transgenic technology that MdPRE4.3 could increase apple sensitivity to NaCl, ABA, and IAA and improve BR tolerance, but not the GA response. Altogether, this study lays the foundation for elucidating the biological and molecular functions of apple’s atypical bHLH transcription factors.

## Data Availability

The original contributions presented in the study are included in the article/Supplementary Material; further inquiries can be directed to the corresponding authors.

## Supplementary Material

Supplementary Figure S1 Evolutionary tree analysis of PRE proteins in apple, *Arabidopsis thaliana*, tomatoes, *Gossypium hirsutum*, and ILI proteins in rice (neighbor-joining tree). Black triangle (▲) represents the MdPRE protein.

## References

[B1] An Y. Zhou Y. Han X. Shen C. Wang S. Liu C. (2019). The GATA Transcription Factor GNC Plays an Important Role in Photosynthesis and Growth in poplar. J. Exp. Bot. 71 (6), 1969–1984. 10.1093/jxb/erz564 PMC7094078 31872214

[B2] Bai M.-Y. Fan M. Oh E. Wang Z.-Y. (2012a). A Triple Helix-Loop-Helix/Basic Helix-Loop-Helix Cascade Controls Cell Elongation Downstream of Multiple Hormonal and Environmental Signaling Pathways inArabidopsis. Plant Cell 24 (12), 4917–4929. 10.1105/tpc.112.105163 23221598 PMC3556966

[B3] Bai M.-Y. Shang J.-X. Oh E. Fan M. Bai Y. Zentella R. (2012b). Brassinosteroid, Gibberellin and Phytochrome Impinge on a Common Transcription Module in Arabidopsis. Nat. Cel Biol 14 (8), 810–817. 10.1038/ncb2546 PMC3606816 22820377

[B4] Bailey T. L. Boden M. Buske F. A. Frith M. Grant C. E. Clementi L. (2009). MEME SUITE: Tools for Motif Discovery and Searching. Nucleic Acids Res. 37. (Web Server issue), W202–W208. 10.1093/nar/gkp335 19458158 PMC2703892

[B5] Bastian R. Dawe A. Meier S. Ludidi N. Bajic V. B. Gehring C. (2010). Gibberellic Acid and cGMP-dependent Transcriptional Regulation inArabidopsis Thaliana. Plant Signaling Behav. 5 (3), 224–232. 10.4161/psb.5.3.10718 PMC2881265 20118660

[B53] Carretero-Paulet L. Galstyan A. Roig-Villanova I. Martínez-García J. F. Bilbao-Castro J. R. Robertson D. L. (2010). Genome-Wide Classification and Evolutionary Analysis of the bHLH Family of Transcription Factors in Arabidopsis, Poplar, Rice, Moss, and Algae. Plant Physiol. 153 (3), 1398–1412. 10.1104/pp.110.153593 20472752 PMC2899937

[B6] Castelain M. Le Hir R. Bellini C. (2012). The Non-DNA-binding bHLH Transcription Factor PRE3/bHLH135/ATBS1/TMO7 Is Involved in the Regulation of Light Signaling Pathway in Arabidopsis. Physiol. Plant 145 (3), 450–460. 10.1111/j.1399-3054.2012.01600.x 22339648

[B18] Chao J. Kong Y. Wang Q. Sun Y. Gong D. Lv J. (2015). MapGene2Chrom, a Tool to Draw Gene Physical Map Based on Perl and SVG Languages. Yi Chuan 37 (1), 91–97. 10.16288/j.yczz.2015.01.013 25608819

[B7] Daccord N. Celton J.-M. Linsmith G. Becker C. Choisne N. Schijlen E. (2017). High-quality De Novo Assembly of the Apple Genome and Methylome Dynamics of Early Fruit Development. Nat. Genet. 49 (7), 1099–1106. 10.1038/ng.3886 28581499

[B8] Feller A. Machemer K. Braun E. L. Grotewold E. (2011). Evolutionary and Comparative Analysis of MYB and bHLH Plant Transcription Factors. Plant Journal 66 (1), 94–116. 10.1111/j.1365-313x.2010.04459.x 21443626

[B9] Gruszka D. (2018). Crosstalk of the Brassinosteroid Signalosome with Phytohormonal and Stress Signaling Components Maintains a Balance between the Processes of Growth and Stress Tolerance. Int. J. Mol. Sci. 19 (9), 2675. 10.3390/ijms19092675 PMC6163518 30205610

[B10] Guo Pengyu Y. Z. (2021). Bioinformatics Analysis of in Tomato, Arabidopsis PREs and Rice ILIs Genes. Janhui Agric. 49 (18), 6. 10.3969/j.issn.0517-6611.2021.18.025

[B11] Hao Y. Oh E. Choi G. Liang Z. Wang Z.-Y. (2012). Interactions between HLH and bHLH Factors Modulate Light-Regulated Plant Development. Mol. Plant 5 (3), 688–697. 10.1093/mp/sss011 22331621 PMC3628346

[B12] Heang D. Sassa H. (2012). Antagonistic Actions of HLH/bHLH Proteins Are Involved in Grain Length and Weight in rice. PLoS One 7 (2), e31325. 10.1371/journal.pone.0031325 22363621 PMC3283642

[B13] Hernandez J. M. Feller A. Morohashi K. Frame K. Grotewold E. (2007). The Basic helix Loop helix Domain of maize R Links Transcriptional Regulation and Histone Modifications by Recruitment of an EMSY-Related Factor. Proc. Natl. Acad. Sci. 104 (43), 17222–17227. 10.1073/pnas.0705629104 17940002 PMC2040437

[B14] Herold S. Wanzel M. Beuger V. Frohme C. Beul D. Hillukkala T. (2002). Negative Regulation of the Mammalian UV Response by Myc through Association with Miz-1. Mol. Cel 10 (3), 509–521. 10.1016/s1097-2765(02)00633-0 12408820

[B15] Hu B. Jin J. Guo A.-Y. Zhang H. Luo J. Gao G. (2015). GSDS 2.0: an Upgraded Gene Feature Visualization Server. Bioinformatics 31 (8), 1296–1297. 10.1093/bioinformatics/btu817 25504850 PMC4393523

[B16] Hyun Y. Lee I. (2006). KIDARI, Encoding a Non-DNA Binding bHLH Protein, Represses Light Signal Transduction in *Arabidopsis thaliana* . Plant Mol. Biol. 61 (1-2), 283–296. 10.1007/s11103-006-0010-2 16786307

[B17] Ikeda M. Mitsuda N. Ohme-Takagi M. (2013). ATBS1 INTERACTING FACTORs Negatively regulateArabidopsiscell Elongation in the Triantagonistic bHLH System. Plant Signaling Behav. 8 (3), e23448. 10.4161/psb.23448 PMC3676513 23333962

[B19] Kelley L. A. Mezulis S. Yates C. M. Wass M. N. Sternberg M. J. E. (2015). The Phyre2 Web portal for Protein Modeling, Prediction and Analysis. Nat. Protoc. 10 (6), 845–858. 10.1038/nprot.2015.053 25950237 PMC5298202

[B20] Kumar S. Stecher G. Li M. Knyaz C. Tamura K. (2018). MEGA X: Molecular Evolutionary Genetics Analysis across Computing Platforms. Mol. Biol. Evol. 35 (6), 1547–1549. 10.1093/molbev/msy096 29722887 PMC5967553

[B28] Laura M. Félix J. M. Francisco J. M. José A. M. Enriqueta M. Antonio R. (2019). An Atypical HLH Transcriptional Regulator Plays a Novel and Important Role in Strawberry Ripened Receptacle. BMC Plant Biol. 19, 586. 10.1186/s12870-019-2092-4 31881835 PMC6933692

[B21] Lee S. Lee S. Yang K.-Y. Kim Y.-M. Park S.-Y. Kim S. Y. (2006). Overexpression of PRE1 and its Homologous Genes Activates Gibberellin-dependent Responses in *Arabidopsis thaliana* . Plant Cel. Physiol. 47 (5), 591–600. 10.1093/pcp/pcj026 16527868

[B22] Letunic I. Bork P. (2018). 20 Years of the SMART Protein Domain Annotation Resource. Nucleic Acids Res. 46 (D1), D493–D496. 10.1093/nar/gkx922 29040681 PMC5753352

[B23] Li J. (2010). Regulation of the Nuclear Activities of Brassinosteroid Signaling. Curr. Opin. Plant Biol. 13 (5), 540–547. 10.1016/j.pbi.2010.08.007 20851039 PMC2967607

[B46] Li X. Duan X. Jiang H. Sun Y. Tang Y. Yuan Z. (2006). Genome-wide Analysis of basic/helix-loop-helix Transcription Factor Family in rice and Arabidopsis. Plant Physiol. 141 (4), 1167. 10.1104/pp.106.080580 16896230 PMC1533929

[B24] Lu R. Zhang J. Liu D. Wei Y.-L. Wang Y. Li X.-B. (2018). Characterization of bHLH/HLH Genes that Are Involved in Brassinosteroid (BR) Signaling in Fiber Development of Cotton (Gossypium Hirsutum). BMC Plant Biol. 18 (1), 304. 10.1186/s12870-018-1523-y 30482177 PMC6258498

[B25] Michal L. L. Chen Y. Israeli A. Efroni I. (2019). Deep Conservation of Response Element Variants Regulating Plant Hormonal Responses. The Plant Cell 31 11. 10.1101/544684 PMC6881130 31467248

[B26] Mao K. Dong Q. Li C. Liu C. Ma F. (2017). Genome Wide Identification and Characterization of Apple bHLH Transcription Factors and Expression Analysis in Response to Drought and Salt Stress. Front. Plant Sci. 8, 480. 10.3389/fpls.2017.00480 28443104 PMC5387082

[B27] Mara C. D. Huang T. Irish V. F. (2010). TheArabidopsisFloral Homeotic Proteins APETALA3 and PISTILLATA Negatively Regulate theBANQUOGenes Implicated in Light Signaling. Plant Cell 22 (3), 690–702. 10.1105/tpc.109.065946 20305124 PMC2861465

[B30] Murre C. Mccaw P. S. Vaessin H. Caudy M. Jan L. Y. Jan Y. N. (1989). Interactions between Heterologous helix-loop-helix Proteins Generate Complexes that Bind Specifically to a Common DNA Sequence. Cell 58 (3), 537–544. 10.1016/0092-8674(89)90434-0 2503252

[B31] Ning L. Wei S. Jing C. Yang F. Kong L. Chu Z. (2017). OsASR2 Regulates the Expression of a Defense-Related Gene. Os2H16, by targeting the GT-1 cis-element. Plant. Biotechnol J. 16, 771–783. 10.1111/pbi.12827 28869785 PMC5814579

[B32] Oh E. Zhu J. Y. Bai M. Y. Arenhart R. A. Sun Y. Wang Z. Y. (2014). Cell Elongation Is Regulated through a central Circuit of Interacting Transcription Factors in the Arabidopsis Hypocotyl. Elife 3, e03031. 10.7554/elife.03031 PMC4075450 24867218

[B33] Oh E. Zhu J. Y. Wang Z. Y. (2012). Interaction between BZR1 and PIF4 Integrates Brassinosteroid and Environmental Responses. Nat. Cel Biol. 14 (8), 802–809. 10.1038/ncb2545 PMC3703456 22820378

[B34] Petroni K. Kumimoto R. W. Gnesutta N. Calvenzani V. Fornari M. Tonelli C. (2012). The Promiscuous Life of Plant NUCLEAR FACTOR Y Transcription Factors. Plant Cell 24 (12), 4777–4792. 10.1105/tpc.112.105734 23275578 PMC3556957

[B35] Porto M. S. Pinheiro M. P. N. Batista V. G. L. dos Santos R. C. de Albuquerque Melo Filho P. de Lima L. M. (2014). Plant Promoters: an Approach of Structure and Function. Mol. Biotechnol. 56 (1), 38–49. 10.1007/s12033-013-9713-1 24122284

[B37] Schlereth A. Moller B. Liu W. Kientz M. Flipse J. Rademacher E. H. (2010). MONOPTEROS Controls Embryonic Root Initiation by Regulating a mobile Transcription Factor. Nature 464 (7290), 913–916. 10.1038/nature08836 20220754

[B38] Sessa G. Morelli G. Ruberti I. (1993). The Athb-1 and -2 HD-Zip Domains Homodimerize Forming Complexes of Different DNA Binding Specificities. Embo J. 12 (9), 3507–3517. 10.1002/j.1460-2075.1993.tb06025.x 8253077 PMC413627

[B39] Shin K. Lee I. Kim E. Park S. K. Soh M. S. Lee S. (2019). PACLOBUTRAZOL-RESISTANCE Gene Family Regulates Floral Organ Growth with Unequal Genetic Redundancy in *Arabidopsis thaliana* . Int. J. Mol. Sci. 20 (4), 869. 10.3390/ijms20040869 PMC6412927 30781591

[B40] Siefers N. Dang K. K. Kumimoto R. W. Bynum W. T. Tayrose G. Holt B. R. (2009). Tissue-specific Expression Patterns of Arabidopsis NF-Y Transcription Factors Suggest Potential for Extensive Combinatorial Complexity. Plant Physiol. 149 (2), 625–641. 10.1104/pp.108.130591 19019982 PMC2633833

[B41] Singh M. Gupta A. Singh D. Khurana J. P. Laxmi A. (2017). Arabidopsis RSS1 Mediates Cross-Talk between Glucose and Light Signaling during Hypocotyl Elongation Growth. Sci. Rep. 7 (1), 16101. 10.1038/s41598-017-16239-y 29170398 PMC5701026

[B42] Tanaka A. Nakagawa H. Tomita C. Shimatani Z. Ohtake M. Nomura T. (2009). BRASSINOSTEROID UPREGULATED1, Encoding a helix-loop-helix Protein, Is a Novel Gene Involved in Brassinosteroid Signaling and Controls Bending of the Lamina Joint in rice. Plant Physiol. 151 (2), 669–680. 10.1104/pp.109.140806 19648232 PMC2754635

[B43] Wang H. Zhu Y. Fujioka S. Asami T. Li J. Li J. (2009). Regulation of Arabidopsis Brassinosteroid Signaling by Atypical Basic helix-loop-helix Proteins. The Plant cell 21 (12), 3781–3791. 10.1105/tpc.109.072504 20023194 PMC2814491

[B44] Waterhouse A. M. Procter J. B. Martin D. M. Clamp M. Barton G. J. (2009). Jalview Version 2--a Multiple Sequence Alignment Editor and Analysis Workbench. Bioinformatics 25 (9), 1189–1191. 10.1093/bioinformatics/btp033 19151095 PMC2672624

[B45] Wei K. Chen H. (2018). Comparative Functional Genomics Analysis of bHLH Gene Family in rice, maize and Wheat. BMC Plant Biol. 18 (1), 309. 10.1186/s12870-018-1529-5 30497403 PMC6267037

[B47] Zhang L. Y. Bai M. Y. Wu J. Zhu J. Y. Wang H. Zhang Z. (2009). Antagonistic HLH/bHLH Transcription Factors Mediate Brassinosteroid Regulation of Cell Elongation and Plant Development in rice and Arabidopsis. Plant Cell 21 (12), 3767–3780. 10.1105/tpc.109.070441 20009022 PMC2814508

[B48] Zhao Q. Ren Y. R. Wang Q. J. Wang X. F. You C. X. Hao Y. J. (2016). Ubiquitination-Related MdBT Scaffold Proteins Target a bHLH Transcription Factor for Iron Homeostasis. Plant Physiologyl 172 (3), 01323. 10.1104/pp.16.01323 PMC5100752 27660166

[B49] Zheng K. Wang Y. Wang S. (2019). The Non-DNA Binding bHLH Transcription Factor Paclobutrazol Resistances Are Involved in the Regulation of ABA and Salt Responses in Arabidopsis. Plant Physiol. Biochem. 172 (3), 39239–39245. 10.1016/j.plaphy.2019.03.026 30921735

[B50] Zheng K. Wang Y. Zhang N. Jia Q. Wang X. Hou C. (2017). Involvement of PACLOBUTRAZOL RESISTANCE6/KIDARI, an Atypical bHLH Transcription Factor, in Auxin Responses in Arabidopsis. Front. Plant Sci. 8, 81813. 10.3389/fpls.2017.01813 PMC5660721 29114256

[B51] Zheng L. (2020). Function Analysis and Mechanism Study of GhPRE1 Gene Regulating Plant Type Development in Gossypium Hirsutum L. Yang Ling: Agronomy College, Northwest A&F University.

[B52] Zhu Z. Chen G. Guo X. Yin W. Yu X. Hu J. (2017). Overexpression of SlPRE2, an Atypical bHLH Transcription Factor, Affects Plant Morphology and Fruit Pigment Accumulation in Tomato. Sci. Rep. 7 (1), 5786. 10.1038/s41598-017-04092-y 28724949 PMC5517572

