# Supplementary material for: Genome-Wide Identification of Apple Atypical bHLH Subfamily PRE Members and Functional Characterization of MdPRE4.3 in Response to Abiotic Stress
Source: Front Genet. 2022 Mar 24;13:846559. doi: 10.3389/fgene.2022.846559 (PMC8987198; doi:10.3389/fgene.2022.846559)
Supplement: Supplementary file 1 [file Table1.DOCX]

**Supplemental Table S1.** Primers for qRT-PCR

| Primer name | Primer sequence (F) | Primer sequence (R) |
| --- | --- | --- |
| *MdPRE2.1* | TTCGAGATAGGCGCTCCAAC | GCAAGTCTCTTGCAGGACCT |
| *MdPRE2.2* | TTCGAGATAGGCGCTCCAAC | GGTCGTCAACCTCTCTGTGT |
| *MdPRE3.1* | GGTTTCAGCATCCACAGTGC | CAGCCGCTCACTTAGATCGT |
| *MdPRE3.2* | ACTGCTACAGGAAACGTGCA | CAGCCGCTCACTTAGATCGT |
| *MdPRE4.1* | CGCTCCTTCCTCAGCTTCAT | CCTCCCTATGCAGCCTCTTT |
| *MdPRE4.2* | TACGCGTAATGCTCCGGTAT | CCTCCCTATGCAGCCTCTTT |
| *MdPRE4.3* | CCTCAGCTTCATCATACGCG | TTCCAAAATGCTCGATGCCGC |
| *MdPRE6.1* | GCGTCGAAGGTTTTGCAAGA | TCGTGGCCAAAAGCTCTGAT |
| *MdPRE6.2* | TCGTCGAGCAGGAACAACAA | GCCTATTGCGAATCTCGGGA |
| *Md18S* | ACACGGGGAGGTAGTGACAA | CCTCCAATGGATCCTCGTTA |
